# Differentiated Neurons Are More Vulnerable to Organophosphate and Carbamate Neurotoxicity than Undifferentiated Neurons Due to the Induction of Redox Stress and Accumulate Oxidatively-Damaged Proteins

**DOI:** 10.3390/brainsci13050728

**Published:** 2023-04-26

**Authors:** Anusha W. Mudyanselage, Buddhika C. Wijamunige, Artur Kocon, Wayne G. Carter

**Affiliations:** 1 School of Medicine, University of Nottingham, Royal Derby Hospital Centre, Uttoxeter Road, Derby DE22 3DT, UK; wijesekara@agri.sab.ac.lk (A.W.M.); buddhikawijamunige@agri.sab.ac.lk (B.C.W.); artek.1993@googlemail.com (A.K.); 2 Faculty of Agricultural Sciences, Sabaragamuwa University of Sri Lanka, Belihuloya 70140, Sri Lanka

**Keywords:** aldicarb, azamethiphos, chlorpyrifos, cholinergic toxicity, developmental neurotoxicity, non-cholinergic mechanisms, pesticides

## Abstract

Organophosphate (OP) and carbamate pesticides are toxic to pests through targeted inhibition of acetylcholinesterase (AChE). However, OPs and carbamates may be harmful to non-target species including humans and could induce developmental neurotoxicity if differentiated or differentiating neurons are particularly vulnerable to neurotoxicant exposures. Hence, this study compared the neurotoxicity of OPs, chlorpyrifos-oxon (CPO), and azamethiphos (AZO) and the carbamate pesticide, aldicarb, to undifferentiated versus differentiated SH-SY5Y neuroblastoma cells. OP and carbamate concentration-response curves for cell viability were undertaken using 3-(4,5 dimethylthiazol-2-yl)-2,5-diphenyl-tetrazolium bromide (MTT) and lactate dehydrogenase (LDH) assays and cellular bioenergetic capacity assessed via quantitation of cellular ATP levels. Concentration-response curves for inhibition of cellular AChE activity were also generated and the production of reactive oxygen species (ROS) was monitored using a 2′,7′-dichlorofluorescein diacetate (DCFDA) assay. The OPs and aldicarb reduced cell viability, cellular ATP levels, and neurite outgrowth in a concentration-dependent fashion, from a threshold concentration of ≥10 µM. Neurotoxic potency was in the order AZO > CPO > aldicarb for undifferentiated cells but CPO > AZO > aldicarb for differentiated cells and this toxic potency of CPO reflected its more extensive induction of reactive oxygen species (ROS) and generation of carbonylated proteins that were characterized by western blotting. Hence, the relative neurotoxicity of the OPs and aldicarb in part reflects non-cholinergic mechanisms that are likely to contribute to developmental neurotoxicity.

## 1. Introduction

Pesticides are utilized for the control and management of pests in agricultural, industrial, and domestic settings. Commercial pesticide usage improves the economic viability of crops and increases food production, but pesticides could impact the health of non-target species [1]. Organophosphate (OP) pesticides are widely used insecticides due to their broad-spectrum activity and relatively low cost [2]. However, the somewhat indiscriminate use of OPs over several decades has produced conditions conducive to the development of insects with pesticide resistance, potential environmental and ecological damage, and the possibility of detrimental effects on human health from both acute and chronic pesticide exposures [1,2,3].

Chlorpyrifos (CPF) has been in use since 1965 and is one of the most extensively employed OP insecticides utilized for crop protection, landscaping pest control, and in domestic settings, due to its potent and broad biocidal nature [4,5]. Metabolic biotransformation of CPF results in desulphuration and the production of chlorpyrifos-oxon (CPO), a metabolite of CPF that is a potent acetylcholinesterase (AChE) inhibitor [2,4,5,6,7]. Inhibition of AChE and the induction of cholinergic toxicity is the recognized mode of action of CPF or CPO, although non-cholinergic mechanisms of toxicity, including induction of redox stress and the adduction of non-cholinesterase targets, have also been documented [1,8,9,10,11,12]. Furthermore, a link has been proposed between subclinical and chronic low-dose exposures to OPs including CPF, particularly prenatally, and subsequent neurodevelopmental and neurobehavioural deficits in children and adults [13,14,15,16].

Azamethiphos is also an OP pesticide (organothiophosphate) but azamethiphos is an oxon and therefore, unlike CPF, does not require hepatic biotransformation. AZO is a widely used synthetic OP insecticide that is bioactive as an AChE inhibitor [17,18]. AZO is also used for the control of ectoparasites in aquacultures including salmon and trout [17,19].

Carbamate insecticides are derivatives of N-methyl carbamic acid. They are widely used in agricultural production, as well as for the protection of human and animal health from insect vector-mediated diseases [20]. Global carbamate pesticide usage is second only to that of OP pesticides as insecticides of choice due to their broad-spectrum biocidal activity. Aldicarb is an oxime methylcarbamate insecticide often soil-applied and used to control insects and nematodes and employed in animal husbandry as an acaricide [21,22,23]. Like OP pesticides, the intended mechanism of action for carbamate insecticides, such as aldicarb, is via inhibition of AChE [20,23,24,25].

However, although AChE inhibition is central to the neurotoxicity of OP and carbamate pesticides, this is unlikely to represent the sole mechanism responsible for the symptomology and disorders that arise from exposure to these chemicals and their metabolites. Their high reactivity accounts for the binding and/or adduction of cellular targets other than cholinesterase enzymes, including neuropathy target esterase (NTE), that can trigger organophosphate-induced delayed neurotoxicity (OPIDN) [1,6,8,9,10,11,18,26,27]. In addition, pesticides can induce cellular redox stress through the production of reactive oxygen species (ROS) and reactive nitrogen species (RNS) [12,26,28]. Pesticide exposure can also deplete the activity of the cellular antioxidant enzymes catalase, superoxide dismutase, and glutathione peroxidase, as well as the levels of glutathione, the major cellular thiol and antioxidant [12,26,28]. ROS are produced in healthy cells under normal metabolic conditions but are present at relatively low levels and scavenged by the antioxidant system; however, sufficient pesticide induction of ROS can potentially overwhelm the antioxidant system, triggering cell death [26,28].

OPs and carbamates may induce neurotoxicity that results in the degeneration of neurons [29,30,31], and for some OPs, such as chlorpyrifos, neurotoxic effects may be particularly damaging if encountered prenatally [13,14,15,16]. An increased vulnerability to developmental neurotoxicity could arise if differentiating and/or recently differentiated neurons are more susceptible to pesticide neurotoxicity. Hence, this study aimed to directly compare the neurotoxicity of the bioactive forms of chlorpyrifos, azamethiphos, and aldicarb to undifferentiated versus differentiated neurons and consider the mechanisms of toxicity.

## 2. Materials and Methods

### 2.1. Chemicals and Reagents

SH-SY5Y human neuroblastoma cells were obtained from the European Collection of Authenticated Cell Culture (ECACC) (ECACC-94030304). Chlorpyrifos-oxon (CPO) diethyl (3,5,6-trichloropyridin-2-yl) phosphate (C_9_H_11_C_l3_NO_4_P, MW = 344.5 g/mol, purity 97.2–99.1%) and Azamethiphos (AZO) (6-chloro-3-(dimethoxyphosphorylsulfanylmethyl)-[1,3]oxazolo[4,5-b]pyridin-2-one), (C_9_H_10_ClN_2_O_5_PS, MW = 324.7 g/mol, purity 95–99.5%) were purchased from Greyhound Chromatography, Birkenhead, UK. Aldicarb ([(E)-(2-methyl-2-methylsulfanylpropylidene)amino] N-methylcarbamate), (C_7_H_14_N_2_O_2_S, MW = 190.27 g/mol, purity 99.5%) was from Chem Service Inc. (West Chester, PA, USA) as supplied by Greyhound Chromatography, Birkenhead, UK. Pesticide stock solutions were prepared at 50 mM in 99.5% pure ethyl alcohol (product 459844, Sigma-Aldrich, Poole, UK). 3-(4,5 dimethylthiazol-2-yl)-2,5-diphenyl-tetrazolium bromide (MTT) (product M5655) isopropanol, and dimethyl sulphoxide (DMSO) (product D8418) were purchased from Sigma-Aldrich, Poole, UK and used for MTT cell viability assays. Acetylthiocholine iodide (ATCI) (product A5751) and 5,5′-Dithiobis-(2-Nitrobenzoic Acid) (DTNB) (product D8130) were purchased from Sigma, Poole, UK and were used for a modified Ellman’s assay adapted for a 96-well plate [32,33]. 2′,7′-dichlorofluorescein diacetate (DCFDA) (D6883, Sigma-Aldrich, Poole, UK), and 30% H_2_O_2_ in H_2_O (H1009, Sigma-Aldrich, Poole, UK) was used as a positive control in the measurements of ROS. Radioimmunoprecipitation assay (RIPA, 20-188, Millipore, Burlington, MA, USA) buffer containing protease inhibitors (04693124001, Roche, Munich, Germany) and phosphatase inhibitor cocktail (P0044, Sigma-Aldrich, Poole, UK) were used for cell lysate preparation. 10 mM 2,4-dinitrophenylhydrazine (DNPH) (D199303, Sigma-Aldrich, Poole, UK) prepared in 2N HCL (231-5957, Scientific Laboratory Suppliers (SLS), Nottingham, UK), trichloroacetic acid (TCA) (T0699, Sigma-Aldrich, Poole, UK), ethyl acetate (270989, Sigma-Aldrich, Poole, UK), and guanidine hydrochloride (50950, Sigma-Aldrich, Poole, UK) were used for the protein carbonyl content (PCC) assays.

### 2.2. Cell Culture

SH-SY5Y cells were grown in a culture medium composed of 43.5% Eagle’s minimum essential medium (EMEM) (M4655, Sigma-Aldrich, Poole, UK) supplemented with 43.5% Ham’s F12 nut mix (217665-029, Gibco, Waltham, MA, USA), 10% heat-inactivated foetal bovine serum (FBS) (F9665, Sigma-Aldrich, Poole, UK), 1% non-essential Amino Acid Solution (M7145, Sigma-Aldrich, Poole, UK), 2 mM glutamine (01077 Life Technologies, Paisley, UK) and 1% penicillin-streptomycin solution containing 10,000 IU penicillium and 10 mg/mL streptomycin (Sigma-Aldrich, Poole, UK) in flasks (T25, 130189, ThermoFisher Scientific, Rochester, UK) at 37 °C with an atmosphere of 5% CO_2_ and 95% humidity, and passaged as required.

SH-SY5Y cells were differentiated as described by Encinas et al. (2000) [34] after seeding onto either poly-D-Lysine hydrobromide (PDL) (50 µg/mL) (P6407, Sigma-Aldrich, Poole, UK) -coated cell cultureware or in 96-well microtitre plates (6005649, Perkin Elmer, Groningen, The Netherlands) with 10% FBS media and after settling, cells were grown to 60% confluency. The following day, the cells were treated with differentiation media (10 µM all-trans retinoic acid (RA) (R26625, Sigma-Aldrich, Poole, UK) in low-serum medium (1% FBS) for 6 days and then treated with 20 ng/mL brain-derived neurotrophic factor (BDNF) (B3795, Sigma-Aldrich, Poole, UK) with low-serum media containing RA for a further 2 days, at which time the cells displayed a fully-differentiated morphology according to Shipley et al. (2016) [35]. All experiments were conducted using passage 13 to prevent any morphological outgrowth of the culture and the potential for genetic drift associated with multiple passaging.

### 2.3. Evaluation of Cell Viability

#### 2.3.1. MTT Assay

The effect of CPO, AZO, or aldicarb (0–200 µM) on the viability of both undifferentiated and differentiated cells was determined by a Thiazolyl Blue Tetrazolium Bromide (MTT) reduction assay [36]. In brief, 3 × 10^4^ cells/well were grown in laminin-coated 96-well clear-bottom tissue culture plates. Undifferentiated cells were challenged with each agent for 24 h within the concentration ranges specified by diluting each compound in the cell culture medium. Differentiated cells that were grown on 96-well plates were treated with each compound diluted in cell culture medium. After incubation with the compounds, spent media was removed and then replaced with the corresponding media for differentiated or undifferentiated cells containing 10% of 5 mg/mL MTT and incubated for 4 h. Wells that only received 10% MTT and respective growth media served as background controls. The formazan crystals generated were suspended in 1:1 DMSO and isopropanol solution and the absorbance of wells read at 570 nm using a spectrophotometer (Multiskan Spectrum, Thermo Electron Corporation, Vantaa, Finland). Experiments were performed in triplicates from which an average was taken, and blank (negative control) values were subtracted. Cell viability was expressed as a percentage of survival relative to treated cells from at least five repeated experiments. The concentration of agent producing a 50% inhibition of cell viability (IC_50_ values) was obtained from the concentration-response curves and expressed as means ± standard error of the mean (SEM).

#### 2.3.2. Lactate Dehydrogenase (LDH) Assay

The production of active extracellular LDH in response to pesticide exposures was measured using an LDH assay kit (ab65393, Abcam, Cambridge, UK) according to the manufacturer’s instructions. After agent treatment (1–200 µM, or vehicle control), 50 µL of spent media was removed and LDH activity was measured spectrophotometrically at 450 nm (Multiskan Spectrum, Thermo Electron Corporation, Finland). Assays were performed in triplicates, with blank values from a negative control subtracted from test values. IC_50_ values were obtained from the concentration-response curves and expressed as means ± standard error of the mean (SEM). Experiments were performed with an n-number of at least five.

#### 2.3.3. Measurement of Intracellular ATP Levels

Undifferentiated and differentiated cells that were grown in 6-well plates were treated with MTT cell viability inhibition concentrations that produced 10, 20, 50, and an 80% loss of cell viability. Intracellular ATP levels were quantified using an ATP luminescence assay kit (ATP Bioluminescence Assay Kit CLS II (product 11 699 695 001, Roche, Germany), according to the manufacturer’s protocol. ATP standards were prepared according to the kit protocol across a concentration range of 1 × 10^−4^ to 1 × 10^−10^ M and after the addition of luciferase reagent to each well, the luminescence was measured using a luminometric plate reader (Thermo Fisher Scientific, Fluoroskan Ascent FC, Vantaa, Finland) using an integration time of 1 s. The ATP content in control and pesticide-treated samples were interpolated from the ATP standard curve. Experiments were performed in triplicates and five individual experiments were undertaken from which a mean was calculated, with blank values subtracted.

### 2.4. Measurements of Neurite Extension in Differentiated Cells

SH-SY5Y cells were seeded at 5 × 10^5^ cells/well in 12-well poly-D-Lysine coated plates and grown for 24 h to ensure cell adhesion. Cells were then treated with pesticides in differentiation media for 24 h. Cell images were captured using a phase contrast microscope (Olympus, DP70, London, UK). Cells were considered differentiated if each neuronal cell contained at least one process that was longer than its cell body. The neurite length from 100 randomly chosen cells were measured in five selected regions of each well using the neurite tracer tool in Image J (Image J 1.49k, National Institute of Health, Bethesda, MD, USA). Results are expressed as mean percentage neurite length (±SEM) relative to vehicle-control treated cells.

### 2.5. Measurements of Acetylcholinesterase (AChE) Activity

Inhibition of AChE was assessed using an Ellman’s assay modified for harvested cells [32,33]. Cells that had been treated with neurotoxicant for 24 h were harvested into ice-cold potassium phosphate buffer pH 8.0 and centrifuged at 13,000× *g* for 5 min at 4 °C. The supernatant was discarded, and the resultant cell pellet was retained and resuspended in 1 mL of potassium phosphate buffer pH 8.0. A volume of 100 µL was then assayed as a 1:1 mixture with ATCI and DTNB. Absorbance of the lysate was immediately measured at 412 nm using a spectrophotometer (Multiskan Spectrum, Thermo Electron Corporation, Finland) in kinetic mode, with readings taken every minute at 37 °C, protected from light, for a total of 10 min. Since the absorbance background increased with time, the associated absorbance of buffer blanks was subtracted at each time point for each data point. Corrected absorbance readings for each treatment were normalized to the means of the vehicle control and AChE activity presented as a percentage relative to the vehicle control.

### 2.6. Measurements of Reactive Oxygen Species

The generation of reactive oxygen species (ROS) were quantified using a 2′,7′-dichlorofluorescein diacetate (DCFDA) assay. Undifferentiated or differentiated cells were seeded in clear-bottom black 96-well plates. Cells were treated with agents at concentrations that reduced cell viability by 10, 20, 50, and 80% (by MTT assay) or treated with vehicle control for 6 h and 24 h. DCFDA at 50 µM was added to each well 30 min before the end of the experiment to allow for cellular incorporation. After the treatments, cells were washed twice with ice-cold PBS and then fluorescence quantified with a 485 nm excitation and 535 nm emission (Thermo Fisher Scientific, Fluoroskan Ascent FC device, ThermoFisher, Finland). Undifferentiated or differentiated cells (with their respective media) were treated for 30 min with 0.5 mM H_2_O_2_ together with 50 µM DCFDA as a positive control for ROS generation, with the values generated set at 100% fluorescence. Six replicate assays were performed for each data point, from which an average was calculated after subtracting blank values generated from media alone with DCFDA.

### 2.7. Cell Lysis and Fractionation

After cell treatments, cells were scraped into 0.5 mL of radioimmunoprecipitation assay (RIPA) buffer containing protease and phosphatase inhibitors. The cell suspension was vortexed thoroughly in the RIPA buffer and then passed through a 28 g needle 25 times to ensure homogenization. Homogenates were stored at −20 °C until required. Thawed homogenates were fractionated by differential centrifugation. Firstly, low-speed centrifugation at 500× *g* for 10 min at 4 °C was performed to pellet the cell debris and nuclear fraction. The supernatant produced was then centrifuged at 23,100× *g* for 40 min at 4 °C to generate a crude cytosolic extract, leaving a pellet that was a membrane-enriched fraction [37].

### 2.8. Protein Quantitation

The quantitation of the protein concentration in cell homogenates was performed using a modified Lowry assay [38]. Bovine serum albumin (BSA) protein standards of 1.25, 2.5, 5, 7.5, and 10 µg (5000206, Bio-Rad, Hertfordshire, UK) were used to generate a standard curve. For a volume of 40 µL of cell lysates or protein standards, 20 µL of Reagent A (500-0113, Bio-Rad, Hertfordshire, UK) was added and then 160 µL of Reagent B (500-0114, Bio-Rad, Hertfordshire, UK) and the samples mixed. After 15 min, spectrophotometric measurements were taken at 740 nm using a SpectraMax plate reader (Multiskan Spectrum, Thermo Electron Corporation, Finland). Protein amounts of unknowns were interpolated from the BSA standard curve.

### 2.9. Quantitation of Protein Carbonyl Content

Quantitation of the levels of cellular protein carbonyl content (PCC) was performed based on published methods [39,40]. Cytosolic extracts from undifferentiated or differentiated cells were challenged with neurotoxicants and extracts prepared as described in Section 2.7. To 500 µg of protein, an equivalent volume of 10 mM 2,4-dinitrophenylhydrazine (DNPH) (prepared in 2N HCl) was added, samples were vortexed and then left in the dark for 1 h at room temperature, with vortex mixing every 10 min. An equivalent volume of ice-cold 20% (*w*/*v*) trichloroacetic acid (TCA) was added and samples were incubated for 15 min on ice. Samples were then spun at 10,000× *g* for 5 min at 4 °C, the supernatant was discarded, and the pellets were washed with 1:1 ethanol:ethyl acetate (*v*/*v*) and vortex mixing. Samples were spun at 10,000× *g* for 5 min at 4 °C and the supernatant discarded. Washing with 20% ice-cold TCA and then 1:1 ethanol:ethyl acetate was repeated and then samples were air-dried for 5 min to allow complete evaporation of solvents. Protein pellets were resuspended in an equal volume of 6 M guanidine hydrochloride in 50 mM phosphate buffer, pH 2.3, with incubation at 37 °C for 30 min and vortex mixing. PCC of test samples was determined via a spectrophotometric reading at 366 nm (Multiskan Spectrum, Thermo Electron Corporation, Finland) using a molar absorption coefficient of 22,000 M^−1^ cm^−1^ after subtraction of blanks. Assays were performed in triplicate from which an average was calculated. A minimum n-number of five was performed for each data point, from which an average was determined.

### 2.10. Characterisation of Carbonylated Proteins Using Oxyblots

Carbonylated proteins produced in response to neurotoxicant treatments were characterized after gel electrophoresis via the use of an OxyBlot Protein Oxidation Detection Kit (S71590, Millipore, Temecula, CA, USA). Cytosolic proteins from pesticide-treated cells were prepared to a concentration of 2 mg/mL and 20 µg derivatized with 2,4-dinitrophenylhydrazine (DNPH) after denaturation with 12% sodium dodecyl sulphate (SDS), according to the manufacturer’s protocol. Samples were neutralized after 15 min and 0.05% of β-mercaptoethanol was added. Proteins were separated by gel electrophoresis using XCell SureLock Mini-Cell Electrophoresis System (E10001, ThermoFisher Scientific, Rochester, UK) and then electroblotted onto polyvinylidene difluoride (PVDF) membranes (88518, ThermoFisher Scientific, Rochester, UK) as previously described [41]. PVDF membranes were dried overnight to fix the proteins. Membranes were rewetted in 10% (*v*/*v*) acetic acid, 50% methanol (*v*/*v*) and 40% (*v*/*v*) ultrapure water for 5 min and then equilibrated with phosphate-buffered saline (PBS) containing 0.05% Tween-20 (PBS-T) washing buffer, blocked for one hour at room temperature with 1% BSA in PBS-T, and then incubated overnight (≈16 h) at 4 °C with a rabbit anti-DNP primary antibody at a 1:150 dilution. Target primary antibody bound to carbonylated proteins was detected using a goat anti-rabbit IgG (HRP-conjugated) secondary antibody at a 1:300 dilution. Detection of immune complexes was accomplished by application of Clarity Western ECL Substrate (BioRad, Hertfordshire, UK), with light captured using a ChemiDoc MP imager (BioRad, Hertfordshire, UK), set for auto-exposure readings to ensure linearity of signal, with representative blots included in Figures.

### 2.11. Statistical Analysis

Results for cell viability and ATP assays are presented as means ± standard error of the mean (SEM), with statistical analysis performed using GraphPad Prism 9.2.0 (GraphPad Prism, San Diego, CA, USA). Concentration-response curves were generated to interpolate the inhibition concentrations in each experiment by using a non-linear regression curve fit model. Curves were plotted using Prism as lines of best fit. Comparison between control and treatment groups was performed using either one-way analysis of variance (ANOVA) or two-way ANOVA with Dennett’s multiple comparison test and Tukey’s multiple comparisons, respectively. A *p* value of <0.05 or lower was considered statistically significant, with asterisks used to indicate levels of significance: * *p* < 0.05; ** *p* < 0.01; *** *p* < 0.001; **** *p* < 0.0001.

## 3. Results

### 3.1. Chlorpyrifos-Oxon, Azamethiphos and Aldicarb Are More Toxic to Differentiated than Undifferentiated Neurons and Reduce Neurite Outgrowth

**Figure 1:**
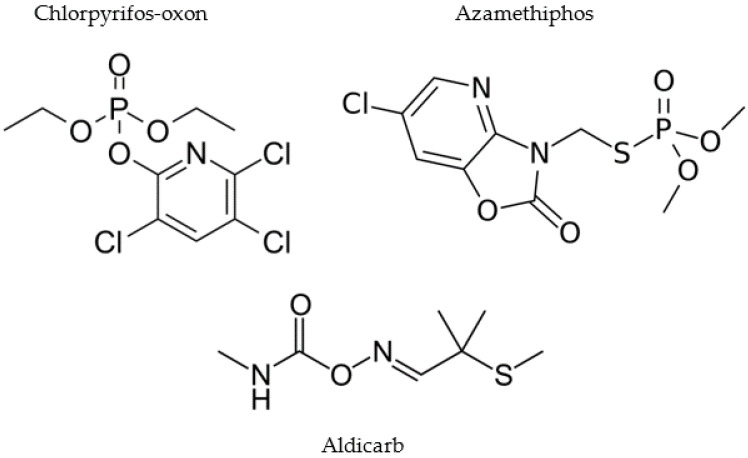
Chemical structures of neurotoxicants investigated in this study.

Undifferentiated and differentiated neuroblastoma SH-SY5Y cells were incubated with chlorpyrifos-oxon (CPO), azamethiphos (AZO), or aldicarb (Figure 1) across a broad concentration range of 1–200 µM for 24 h and cell metabolic activity and viability quantified using an MTT assay. CPO was applied directly to neuronal cells to represent the biologically active metabolite of chlorpyrifos (CPF) that acts as a potent AChE inhibitor.

**Figure 2:**
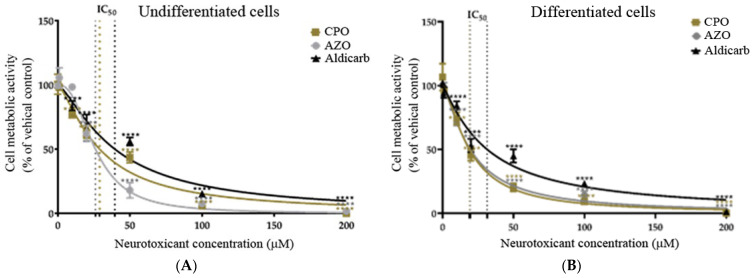
Toxicity of CPO, AZO, and aldicarb to undifferentiated and differentiated SHSY-5Y cells measured using an MTT assay. Undifferentiated (**A**) or differentiated (**B**) SHSY-5Y cells were treated with CPO, AZO, or aldicarb and cell metabolic activity was quantified using an MTT assay. Absorbance readings were normalised to the viability of vehicle controls, providing cell viability as a percentage relative to the vehicle control. Readings were taken from five individual experiments with three replicates in each treatment concentration. Results were analysed using one-way ANOVA with Dunnett’s multiple comparison tests and expressed as mean ± standard error of the mean (SEM). For significance, **** *p* < 0.0001.

Cell viability of SH-SY5Y cells was reduced after OP or aldicarb exposure in a concentration-dependent manner, with differentiated neurons more sensitive to exposures with reduced cell viability at lower concentrations (refer to Figure 2A,B). The concentrations that reduced cell viability by 50% (IC_50_ values) were calculated by non-linear regression and values have been included in Table 1. The threshold for a significant reduction of cell viability for both cell phenotypes was a neurotoxicant concentration of ≥10 µM.

**Figure 3:**
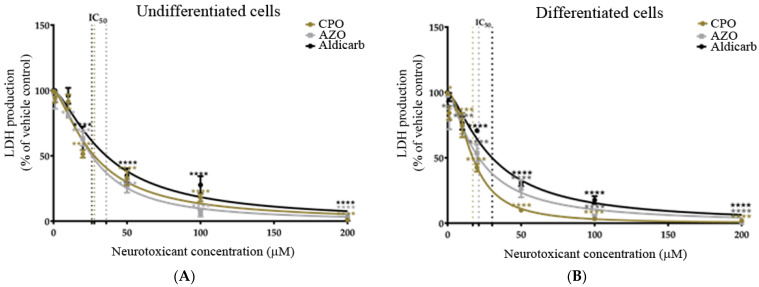
Toxicity of CPO, AZO, and aldicarb to undifferentiated and differentiated SHSY-5Y cells measured using a LDH assay. Undifferentiated (**A**) or differentiated (**B**) SHSY-5Y cells were treated with CPO, AZO, or aldicarb and extracellular LDH production was quantified using an LDH activity assay. Absorbance readings were corrected by subtracting the values of blanks, then the resultant values were normalised to the LDH production from a vehicle control, providing LDH production as a percentage value relative to the vehicle control. Readings were taken from five independent experiments with three replicates for each treatment. Results were analysed using one-way ANOVA with Dunnett’s multiple comparison tests and expressed as means ± standard error of the mean (SEM). For significance, *** *p* < 0.001, **** *p* < 0.0001.

As an alternative measure of cell viability, the production of active, extracellular LDH was quantified after 24-h exposure to the neurotoxicants. Undifferentiated or differentiated cells displayed a concentration-dependent loss of cell viability (Figure 3A,B), with a significant reduction of viability from agent concentrations of ≥10 µM. Similar to the MTT data, the OPs (CPO and AZO) were more toxic than aldicarb to both cell phenotypes (lower IC_50_ values) and the potency of toxicity decreased in the order AZO > CPO > aldicarb in undifferentiated cells but CPO > AZO > aldicarb for differentiated cells. Furthermore, all compounds were more neurotoxic to differentiated cells than undifferentiated cells, with lower IC_50_ values (refer to Table 1).

**Figure 4:**
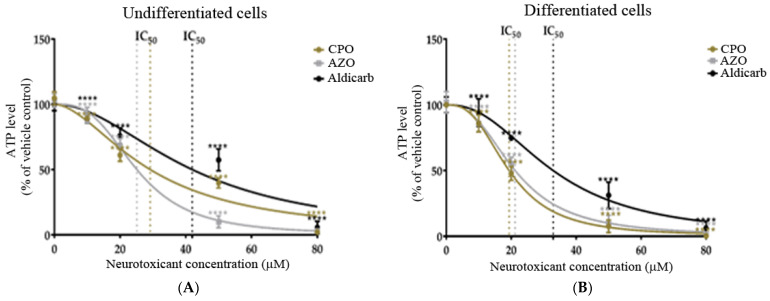
Toxicity of CPO, AZO, and aldicarb to undifferentiated and differentiated SHSY-5Y cells measured using an ATP assay. Undifferentiated (**A**) or differentiated (**B**) SHSY-5Y cells were treated with CPO, AZO, or aldicarb and intracellular ATP levels were quantified using an ATP assay. Inhibition concentrations were interpolated from MTT assays with IC_10_, IC_20_, IC_50,_ and IC_80_ concentrations selected for ATP assays. Absorbance readings were corrected by subtracting blank values with the resultant values normalised to the ATP level of vehicle controls, providing ATP levels as a percentage value relative to the vehicle control. Readings were taken from five individual experiments with three replicates quantified for each treatment. Results were analysed using one-way ANOVA with Dunnett’s multiple comparison tests and expressed as means ± standard error of the mean (SEM). For significance, **** *p* < 0. 0001.

We next investigated the effects of neurotoxicants on cellular bioenergetics via the quantitation of intracellular ATP levels. CPO, AZO, and aldicarb significantly decreased ATP levels in proportion to their applied concentrations in both SHSY-5Y cell phenotypes (Figure 4A,B). The induced reductions of ATP levels generated IC_50_ values similar to those from MTT and LDH assays (refer to Table 1).

**Figure 5:**
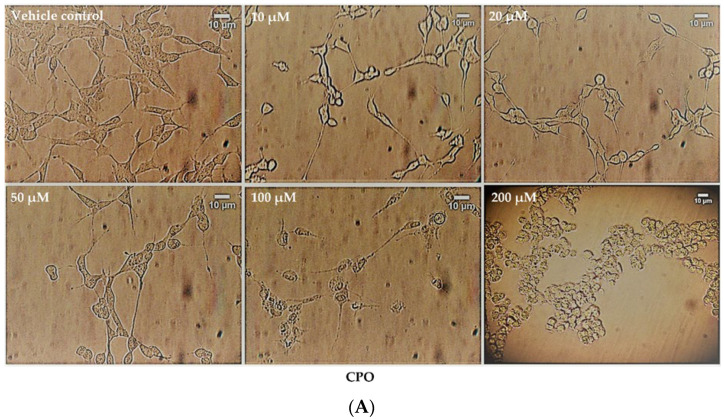

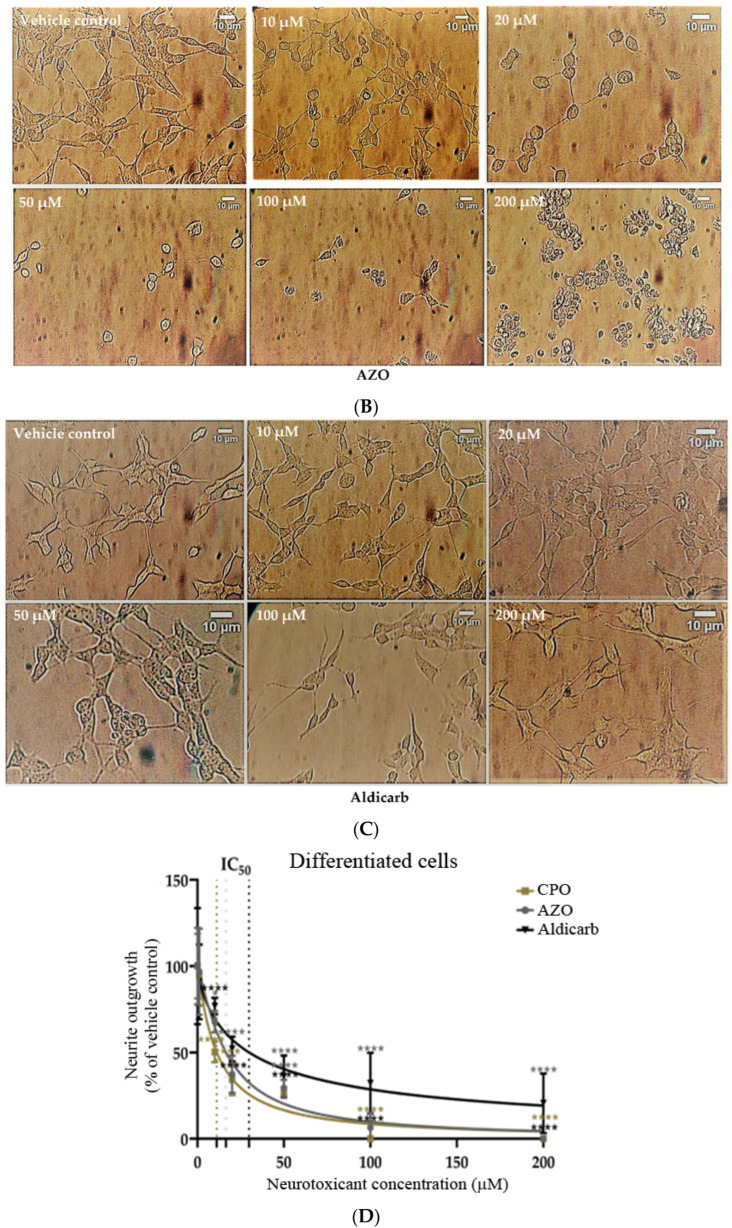
Toxicity of CPO, AZO, and aldicarb to differentiated SHSY-5Y cells assessed using microscopy. SH-SY5Y cells were induced to differentiate for 24 h and then treated with CPO (**A**), AZO (**B**), or aldicarb (**C**) over a concentration range of 0–200 µM and morphological changes were captured using phase-contrast microscopy. Neurite lengths were quantified using the neurite tracer tool in ImageJ (National Institute of Health, USA) from five independent experiments with three replicates (**D**). Results were analysed using one-way ANOVA with Dunnett’s multiple comparison tests and expressed as means ± standard error of the mean (SEM). For significance, **** *p* < 0. 0001.

The effect of the neurotoxicants on cell number and cell morphology was further evaluated by bright-field, phase-contrast microscopy. Cell numbers declined in proportion to increasing agent concentration and neurotoxicants also reduced the levels of neurite outgrowth in pre-differentiated cells in a concentration-dependent manner (Figure 5A–D). At the higher agent concentrations, cells adopted a rounded morphology, particularly after CPO or AZO treatments, but some cells survived and were resistant to cell death even at the highest pesticide concentrations examined (200 µM). CPO was the most potent inhibitor of neurite retraction followed by AZO and then aldicarb (refer to Table 1).

**Figure 6:**
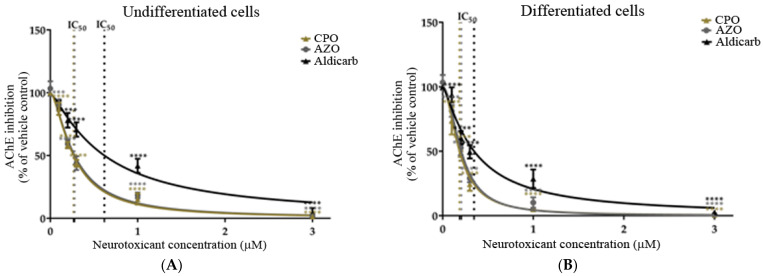
Toxicity of CPO, AZO, and aldicarb to undifferentiated and differentiated SHSY-5Y cells measured via inhibition of AChE. Undifferentiated (**A**) or differentiated (**B**) SHSY-5Y cells were treated with CPO, AZO, or aldicarb over the concentration range of 0–3 µM for 24 h and the inhibition of AChE quantified using a modified Ellman’s assay. Assay absorbance readings were normalised to vehicle control after subtracting blank values and expressed as a percentage of vehicle controls. Readings were obtained from five individual experiments with three replicates measured for every data point. Results were analysed using one-way ANOVA with Dunnett’s multiple comparison tests and expressed as means ± standard error of the mean (SEM). For significance, **** *p* < 0.0001.

Cholinergic toxicity to SHSY-5Y cells was considered through analysis of the inhibition of endogenous AChE. Undifferentiated and differentiated cells were incubated with the neurotoxicants for 24 h over a concentration range of 0–3 µM (range determined by preliminary experiments; results not included) and the inhibition of AChE quantified using a modified Ellman’s assay [32,33] (Figure 6A,B). The concentration of agent that produced 50% inhibition of AChE (IC_50_) was calculated by non-linear regression and values have been included in Table 1. The neurotoxicants were more potent inhibitors of AChE in differentiated than undifferentiated cells, with lower IC_50_ values.

### 3.2. Chlorpyrifos-Oxon, Azamethiphos and Aldicarb Induced Production of Reactive Oxygen Species and Oxidatively-Damaged Proteins

**Figure 7:**
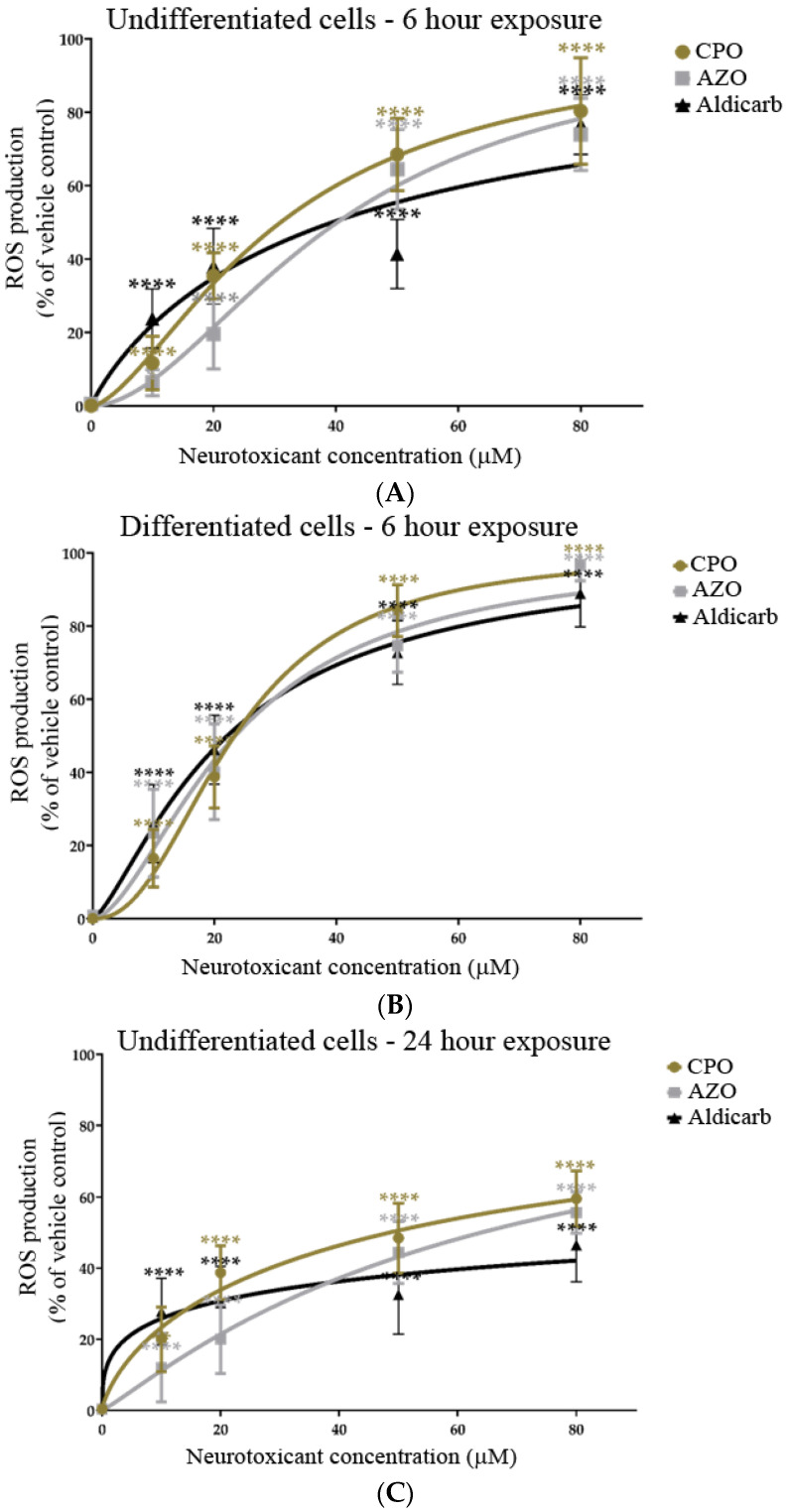

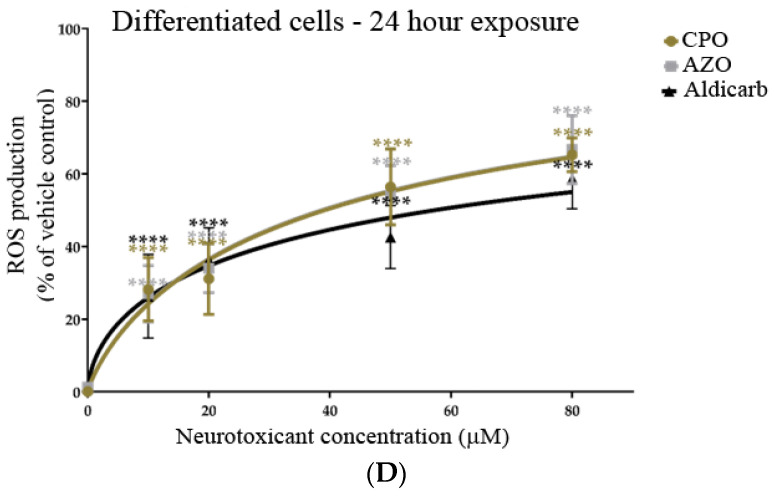
Toxicity of CPO, AZO, and aldicarb to undifferentiated and differentiated SHSY-5Y cells measured using a DCFDA assay. Undifferentiated (**A**,**C**) or differentiated (**B**,**D**) SHSY-5Y cells were treated with CPO, AZO, or aldicarb (MTT IC_10_, IC_20_, IC_50,_ and IC_80_ concentrations) and ROS levels were quantified using a DCFDA assay after 6 or 24 h. Cellular ROS levels were normalised to vehicle control treatments and presented as a percentage of vehicle control. Results were obtained from five individual experiments with three replicates measured for every data point. Results were analysed using one-way ANOVA with Dunnett’s multiple comparison tests and expressed as means ± standard error of the mean (SEM). For significance, **** *p* < 0.0001.

The induction of reactive oxygen species (ROS) in response to treatment of SHSY-5Y cells with CPO, AZO, or aldicarb after 6 and 24 h was tracked using a DCFDA assay. ROS levels were quantified relative to the levels produced by 500 µM H_2_O_2_ as a positive control cellular redox stressor. ROS levels within undifferentiated or differentiated cells increased significantly after 6 h, in accordance with agent concentration, and plateaued at 50–80 µM (Figure 7A,B). Similarly, a 24-h exposure to the neurotoxicants induced ROS levels that increased in proportion to their concentrations and plateaued at 50–80 µM (Figure 7C,D). Collectively, ROS levels were significantly higher in differentiated cells than in undifferentiated cells at high pesticide concentrations and were associated with reduced cell viability (refer to Supplementary Data). ROS levels were higher at 6 than 24 h and, for the higher neurotoxicant concentrations, were produced in the order CPO > AZO > Aldicarb (Figure 7A–D).

**Figure 8:**
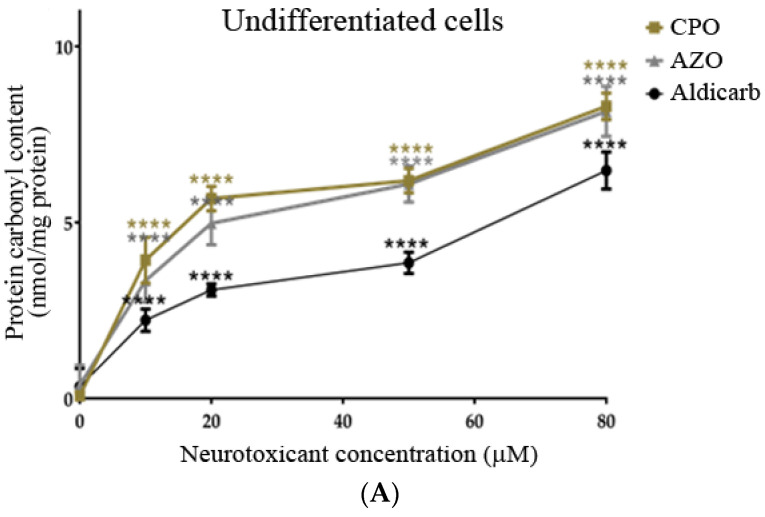

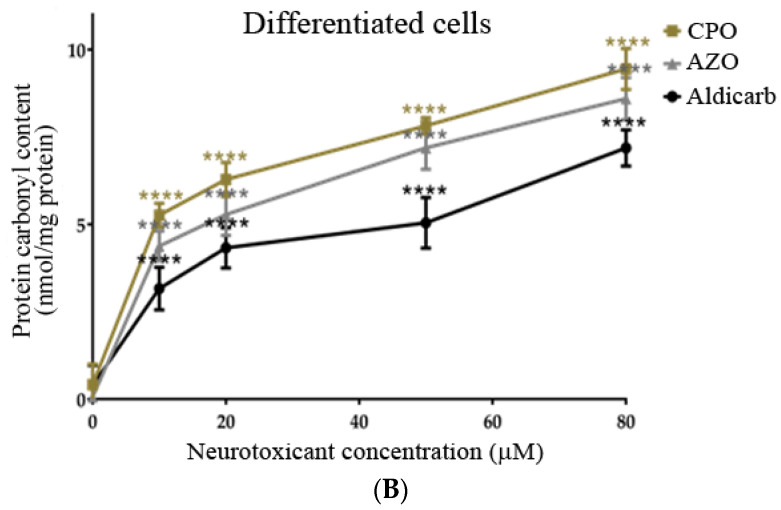
CPO, AZO, and aldicarb induction of protein carbonyl content in undifferentiated and differentiated SHSY-5Y cells. Undifferentiated (**A**) or differentiated (**B**) SHSY-5Y cells were treated with CPO, AZO, or aldicarb and the levels of protein carbonyl content (PCC) were quantified after 24 h. PCC was quantified after DNPH derivatization of the cytosolic fractions of treated cells and spectrophotometric readings normalised to vehicle control treatments after subtracting the blanks. Triplicates assays were performed for each data point and five individual experiments were undertaken. Results were analysed using one-way ANOVA with Dunnett’s multiple comparison tests and expressed as means ± standard error of the mean (SEM). For significance, **** *p* < 0.0001.

The levels of protein carbonyl content (PCC) (oxidatively-damaged proteins) in response to a 24-h exposure to CPO, AZO, or aldicarb were quantified. All three agents significantly increased PCC in a concentration-dependent manner in both undifferentiated and differentiated cells (Figure 8A,B). PCC levels mirrored those of ROS such that induction was in the order CPO > AZO > aldicarb (Figure 8A,B), with levels significantly higher in differentiated cells compared to undifferentiated cells (refer to Supplementary Data).

**Figure 9:**
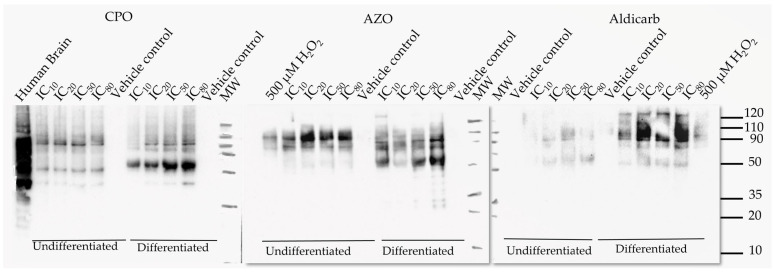
CPO, AZO, and aldicarb induction of carbonylated proteins in undifferentiated and differentiated SHSY-5Y cells characterized by oxy-blotting. Undifferentiated or differentiated SHSY-5Y cells were treated with CPO, AZO, or aldicarb at their MTT IC10, IC20, IC50, and IC80 concentrations and carbonylated proteins detected using an oxy-blot. Three independent blotting experiments were performed with each pesticide with representative blots included.

To characterise the oxidatively-damaged proteins, cytosolic proteins from undifferentiated and differentiated SH-SY5Y cells that had been treated with the neurotoxicants were resolved by gel electrophoresis and carbonylated proteins localised by immune (oxy)-blotting (Figure 9).

The most prominent and reproducible carbonylated proteins were characterized by their denatured molecular weights of approximately 35, 50, 80, and 90 kDa. The accumulation of oxidatively-damaged proteins primarily increased in proportion to agent concentration, most notably the ≈50 kDa protein.

Collectively, differentiated cells were more vulnerable to the neurotoxic effects of the OPs and aldicarb and this was in part mediated through the induction of redox stress as a non-cholinergic mechanism.

## 4. Discussion

### 4.1. Differentiated Neurons Are More Vulnerable to the Neurotoxic Effects of CPO, AZO, and Aldicarb

In this manuscript, the neurotoxic properties of two OPs, CPO and AZO, and the carbamate pesticide, aldicarb, were investigated. All three compounds were cytotoxic and reduced neuronal viability and were detrimental to cellular bioenergetics (ATP production) (Figure 2, Figure 3 and Figure 4). Neurotoxicity potency was in the order AZO > CPO > aldicarb for undifferentiated cells but CPO > AZO > aldicarb for differentiated cells. Vulnerability to neurotoxicity for differentiated cells was evidenced through suppression of neurite outgrowth (Figure 5), increased inhibition of AChE (Figure 6), and increased production of damaging ROS (Figure 7). CPO induced the highest levels of ROS and corresponding production of oxidatively-damaged proteins, and these were higher in differentiated cells than undifferentiated cells and were characterized by their denatured molecular weights (Figure 8 and Figure 9).

SH-SY5Y cells were used as a homogeneous neuronal cell model, often used for toxicity studies, since they can be specifically differentiated to undergo a morphological change and generate neuritic projections, with altered neurotransmitter responsiveness consistent with a cholinergic phenotype [34,35,42,43,44]. Through a direct comparison of undifferentiated with differentiated SH-SY5Y cells, an increased vulnerability of differentiated cells to neurotoxicants was established. MTT assays provided a measurement of cell metabolic activity as a surrogate for cell viability, with the neurotoxicant-induced loss of cell viability substantiated using LDH assays and a shutdown of cellular ATP production (Figure 2, Figure 3 and Figure 4).

### 4.2. Differentiated Neurons Are More Vulnerable to Cholinergic Toxicity from CPO, AZO, and Aldicarb

The recognized acute neurotoxicity of many OPs and carbamates is via the targeted inhibition of AChE and the induction of cholinergic crisis. By challenging cells with the bioactive oxon forms of the OP pesticides (CPO and AZO) and aldicarb, we were able to consider their relative potency as cholinesterase inhibitors. All three compounds were strong inhibitors of AChE with IC_50_ values of below 1 µM, but the OPs were more potent than aldicarb, with CPO displaying the lowest IC_50_ concentration (Figure 6 and Table 1). A key difference between the AChE inhibition by the two OPs and aldicarb is the stability of the AChE-OP and the AChE–carbamate complex, since the organophosphylation of AChE is often stable with slow spontaneous hydrolysis (particularly for O,O’-diethyl adducts such as those produced from CPO), whereas carbamylation at the active site serine of AChE is readily reversible and can spontaneously hydrolyse within hours [23]. Furthermore, carbamate binding to AChE does not undergo non-enzymatic dealkylation (ageing) which can further limit hydrolysis of the organophosphorylation such that it becomes essentially irreversible. Thus, if the potential for prolonged inhibition of AChE is solely considered, aldicarb should be considered less toxic than either CPO or AZA. However, the potentially rapid binding of aldicarb to AChE and associated acute toxicity (rat oral LD_50_ of 0.65 mg/kg) [45] without the need for bioactivation, has resulted in aldicarb (and its formulations) being classified as extremely hazardous (Class Ia) in contrast to the moderately hazardous listing for chlorpyrifos (CPF) and Azamethiphos (AZO) (Class II) (rat oral LD_50_ of 82 mg/kg and 1180 mg/kg, respectively) [46,47,48].

The production of AChE inhibitor response curves for treatments with CPO, AZO, and aldicarb revealed that differentiated neurons were more vulnerable to neurotoxicity, with lower IC_50_ values (by 27–39%). Collectively, this neurotoxicant inhibition of AChE occurs at lower concentrations (IC_50_ values of 0.19–0.61 µM) than those investigated for ROS production. However, consideration should be given to the combination of both mechanisms of toxicity since inhibition of AChE may be transient. AZO produces O,O’-dimethyl adducts with AChE which are less stable than the O,O’-diethyl adducts produced from CPO, and can be hydrolyzed after several hours. Nevertheless, irrespective of whether pesticide-AChE O,O’-dimethyl or O,O’-diethyl adducts are formed, AChE will be regenerated through protein turnover, with an estimated half-life of 3–12 days [49], and this may be even more rapid for an irreversible inhibitor-bound enzyme [49]. Furthermore, AChE has a rapid enzymatic turnover number [50]; hence, even low levels of the enzyme could still potentially cleave ACh efficiently, assuming the substrate is not limiting. In addition, relatively high (or repeated) exposures to OP inhibitors are usually needed to surpass the threshold of approximately 50–75% AChE inhibition associated with mild to moderate OP poisoning [51]. By contrast, the structure-activity relationships of other proteins adducted by pesticides may differ from that for AChE [1,6,8,9,10,11,18,26,27,52] and some could potentially be long-lasting and contribute to pesticide-induced ill health. Furthermore, as proposed herein, oxidatively-damaged proteins are generated after exposure to neurotoxicants at a concentration below the threshold required to induce neuronal death, and therefore this form of protein post-translational modification could potentially contribute to acute or chronic cellular damage.

### 4.3. Differentiated Neurons Are More Vulnerable to Non-Cholinergic Toxicity from CPO, AZO, and Aldicarb

We also examined the non-cholinergic mechanism of neurotoxicant-generated redox stress through the induction of ROS and the production of carbonylated proteins. After cell treatment, differentiated cells produced significantly more ROS than their undifferentiated counterparts and hence, significantly higher levels of PCC (refer to Supplementary Data). The induction of PCC was evidenced by the detection and characterization of oxidatively-damaged proteins and herein we provide the first characterization of these carbonylated proteins in response to CPO, AZO, and aldicarb. The most prominent detection of carbonylation was at denatured molecular weights of approximately 35, 50, 80, and 90 kDa. The greater levels of oxidative damage to these proteins, particularly the ≈50 kDa protein band, may represent an increased vulnerability to oxidative damage as a consequence of their higher protein expression in differentiation cells.

SH-SY-5Y cell differentiation is characterized by the expression of mature neuronal markers including βIII-tubulin, microtubule-associated protein-2 (MAP-2), and MAP-tau; cytoskeletal or cytoskeletal-associated proteins required for the production of neurite projections [34,35,42,43,44,53]. The denatured molecular weights of immunoreactive tubulin, MAP-2 and MAP-tau [54] could correspond to the oxidatively-damaged protein bands at approximately 50, 80, and 90 kDa (Figure 9). In support of this supposition, other studies have documented the vulnerability of α- and β-tubulin proteins (of ≈50 kDa) to either organophosphorylation or oxidation [55,56,57], but confirmation of the identity of the major carbonylated proteins that accumulate in response to these neurotoxicants will require future study. Furthermore, future studies will need to consider the fate of these oxidatively-damaged proteins. If the levels of ROS overwhelm the cellular antioxidant system, cell death is triggered [26,28]. However, for cells that survive a neurotoxic insult, these carbonylated proteins may be detrimental to cellular functionality [58] and therefore represent a pesticide-induced cellular injury.

Other studies have confirmed increased carbonylation in response to CPF treatment in neuronal cells or tissue [59,60] but this is the first report of a similar response from AZO and aldicarb. Furthermore, our data suggest that the target proteins for carbonylation may overlap for each of the neurotoxicants, indicative of a common mechanism of neurotoxicity (Figure 9). Carbonylated damage to proteins may contribute to the blunting of neuritic arborization we detected and is consistent with reports that exposure to certain (but not all) pesticides, including AZO, can inhibit neurite outgrowth in vitro [61]. However, in vitro studies, such as ours, are limited by the application of pesticides or their metabolites directly to cells and further in vivo studies are required to substantiate these research findings.

### 4.4. Cholinergic and Non-Cholinergic Toxicity: Considerations for Neurodevelopmental Effects, Treatment of Exposures, and Pesticide Risk Assessment

The susceptibility of neurons to oxidative damage during neurogenesis and neuronal arborization has implications for the impact of pesticide exposures on the developing brain. Studies have indicated that prenatal and childhood exposures to chlorpyrifos can induce neurodevelopmental and neurobehavioural deficits in children and adults [13,14,15,16,62] and this could in part reflect oxidative damage to key proteins involved in the production of neuritic projections and connections between neurons.

Noteworthy is that acute intoxication from OP pesticides is usually through self-harm and is estimated to account for approximately 100,000 deaths per year [63]. The current treatment of OP pesticide exposure utilizes a muscarinic antagonist (typically atropine) and, depending upon the type of OP pesticide poisoning (if known), an oxime (such as pralidoxime) as an AChE reactivator [64]. However, given the importance of non-cholinergic toxicity mediated via redox stress, it would be prudent to also consider treatment with antioxidant co-therapy. Hence, a pesticide risk assessment should consider acute and potential long-lasting toxicity and the clinical sequelae that reflects both cholinergic and non-cholinergic mechanisms.

## Figures and Tables

**Table 1:** Toxicity of CPO, AZO, and aldicarb to undifferentiated and differentiated SH-SY5Y cells.

Cell type	Agent	MTT		LDH		ATP		Neurite Retraction		AChE Inhibition	
		IC_50_	R^2^	IC_50_	R^2^	IC_50_	R^2^	IC_50_	R^2^	IC_50_	R^2^
Undifferentiated	CPO	29.4 ± 2.1	0.970	27.8 ± 2.1	0.971	29.6 ± 2.1	0.956	N/A	-	0.28 ± 0.0	0.996
Differentiated		17.3 ± 0.9	0.987	17.2 ± 1.1	0.967	18.8 ± 0.1	0.999	10.8 ± 0.1	0.914	0.19 ± 0.0	0.987
Undifferentiated	AZO	26.9 ± 1.1	0.987	26.5 ± 0.9	0.992	26.0 ± 0.6	0.995	N/A	-	0.30 ± 0.0	0.992
Differentiated		19.6 ± 0.5	0.996	20.5 ± 1.6	0.954	20.7 ± 0.1	0.999	16.5 ± 1.3	0.894	0.22 ± 0.0	0.986
Undifferentiated	Aldicarb	39.6 ± 3.6	0.951	35.4 ± 1.4	0.990	40.2 ± 2.9	0.945	N/A	-	0.62 ± 0.0	0.967
Differentiated		31.6 ± 2.2	0.973	29.9 ± 1.6	0.981	32.4 ± 1.2	0.987	30.2 ± 4.7	0.703	0.38 ± 0.0	0.970

The concentrations (µM) of the neurotoxicants that produced 50% assay inhibition (IC_50_) are displayed, interpolated from concentration-response curves, and expressed as means ± 95% confidence intervals from five independent experiments. R^2^ values were calculated after graph plotting in Prism to provide a numerical quantitation of how the curve fits the expected non-linear model. AChE, acetylcholinesterase; ATP, adenosine triphosphate; LDH, lactate dehydrogenase; MTT, (4,5-dimethylthiazol-2-yl)-2,5-diphenyltetrazolium bromide; N/A, not applicable.

## Data Availability

Data is available on request from the first or last authors of the manuscript.

## Supplementary Materials

The following supporting information can be downloaded at: https://www.mdpi.com/article/10.3390/brainsci13050728/s1, supplementary data, Table S1: comparison of the cytotoxicity induced by CPO, AZO, and aldicarb to undifferentiated versus differentiated SH-SY5Y cells.
