# Supplementary material for: Differentiated Neurons Are More Vulnerable to Organophosphate and Carbamate Neurotoxicity than Undifferentiated Neurons Due to the Induction of Redox Stress and Accumulate Oxidatively-Damaged Proteins
Source: Brain Sci. 2023 Apr 26;13(5):728. doi: 10.3390/brainsci13050728 (PMC10216341; doi:10.3390/brainsci13050728)
Supplement: Supplementary file 1 [file brainsci-13-00728-s001.zip › brainsci-2358487-supplementary.pdf]

Supplementary Table S1. Comparison of the cytotoxicity induced by CPO, AZO and Aldicarb to undifferentiated versus differentiated SH-SY5Y cells.

Pesticide	Treatments	MTT assay	LDH assay	Treatments	ATP assay	ROS assay		PCC assay
CPO	1	NS	0.0216			6 hours	24 hours	
	10	NS	0.0002	IC_{10}	NS	0.0022	0.0002	<0.0001
	20	0.0005	NS	IC_{20}	0.0031	NS	NS	0.0004
	50	<0.0001	<0.0001	IC_{50}	<0.0001	<0.0001	0.0002	<0.0001
	100	NS	0.0008	IC_{80}	NS	<0.0001	0.0012	<0.0001
	200	NS	NS					
AZO	1	0.0253	0.0056					
	10	<0.0001	NS	IC_{10}	NS	<0.0001	<0.0001	<0.0001
	20	0.0006	0.0145	IC_{20}	0.0444	<0.0001	<0.0001	<0.0001
	50	NS	NS	IC_{50}	NS	<0.0001	<0.0001	<0.0001
	100	NS	NS	IC_{80}	0.0270	<0.0001	<0.0001	<0.0001
	200	NS	NS					
Aldicarb	1	NS	NS					
	10	NS	<0.0001	IC_{10}	NS	NS	NS	<0.0001
	20	0.0361	NS	IC_{20}	NS	0.0006	0.0066	<0.0001
	50	0.0015	NS	IC_{50}	0.0005	<0.0001	<0.0001	<0.0001
	100	NS	NS	IC_{80}	NS	<0.0001	<0.0001	0.1945
	200	NS	NS					

Undifferentiated or differentiated SH-SY5Y cells were treated with chlorpyrifos-oxon (CPO), Azamethiphos-oxon (AZO) or aldicarb. Cell viability measurements were made using MTT, LDH or ATP levels and the production of ROS and PCC was quantified. Comparisons between assays means were made by a two-way ANOVA with Tukey's multiple comparison tests and the values shown for significant changes are marked or listed as NS for non-significant.

Abbreviations: ATP, Adenosine triphosphate; IC, inhibitor concentration (producing 10-80% inhibition); LDH, Lactate dehydrogenase; MTT, (4,5-dimethylthiazol-2-yl)-2,5-diphenyltetrazolium bromide; PCC, protein carbonyl content; ROS, reactive oxygen species.
